# Impact of Zinc Oxide on the Development of *Aspergillus*-Induced Maxillary Sinusitis Rabbit Model

**DOI:** 10.3390/cimb46060342

**Published:** 2024-06-07

**Authors:** Seung-Heon Shin, Mi-Kyung Ye, Dong-Won Lee, Mi-Hyun Choi

**Affiliations:** Department of Otolaryngology-Head and Neck Surgery, School of Medicine, Daegu Catholic University, Daegu 42472, Republic of Korea; miky@cu.ac.kr (M.-K.Y.); neck@cu.ac.kr (D.-W.L.); leonen@hanmail.net (M.-H.C.)

**Keywords:** *Aspergillus fumigatus*, rabbit, fungal sinusitis, inflammation, biofilm

## Abstract

*Aspergillus fumigatus* is commonly found in the airway and is associated with airway inflammatory diseases. Zinc oxide (ZO) is known to be an essential microelement that facilitates fungal survival, growth, and proliferation. This study aimed to investigate the impact of ZO on *A. fumigatus*-induced fungal sinusitis in rabbits. Twenty-eight New Zealand white rabbits were divided into four groups for this study. Group 1 (6 sides) was treated with intramaxillary phosphate buffer saline (PBS) served as the negative control, Group 2 (6 sides) received intramaxillary PBS and ZO, Group 3 (8 sides) was treated with intramaxillary *A. fumigatus* alone, and Group 4 (8 sides) treated with intramaxillary *A. fumigatus* with ZO. After 4 and 12 weeks, sinus mucosal cytokine and transcription factor expressions were determined. A histological analysis was performed to determine inflammatory cell infiltration, number of secretory cells, and mucosal thickness. Fungal biofilm formation was determined using confocal laser microscopy. The intramaxillary instillation of *A. fumigatus* conidia led to an increase in protein and mRNA expression of interleukin (IL)-1β and IL-8 in the maxillary sinus mucosa. They were associated with mitogen-activated protein kinase and activator protein-1. Furthermore, intramaxillary instillation of fungal conidia resulted in significant enhancement of inflammatory cell infiltration, epithelial thickening, and fungal biofilm formation. However, intramaxillary ZO did not have a significant impact on *A. fumigatus*-induced cytokine protein and mRNA expression, and inflammatory cell infiltration and epithelial thickness in sinonasal mucosa. While intramaxillary instillation of *A. fumigatus* increased mucosal inflammation, cytokine production, and biofilm formation, the intramaxillary application of ZO did not have a significant influence on inflammation in the maxillary sinus mucosa.

## 1. Introduction

Chronic rhinosinusitis (CRS) is sinonasal mucosal inflammation caused by abnormal innate and adaptive immune responses against environmental pathogens. Fungi are ubiquitous in the environment, and they are continuously inhaled and deposited in the airway mucosa. They are commonly found in nasal secretions of both CRS and normal healthy volunteers and the role of fungi in the pathogenesis of CRS still remains under debate [1,2]. Nonetheless, many researchers reported abnormal mucosal immune responses against airborne fungi, which can trigger various inflammatory responses in the respiratory mucosa. Among several airborne fungi, Aspergillus species are most commonly associated with airway fungal disease and *Aspergillus fumigatus* is the main cause of fungal rhinosinusitis and sinus fungal ball [2,3]. In CRS, bacterial and fungal biofilms commonly coexist in the sinonasal mucosa [4]. In healthy mucosa with an intact innate immune defense system, fungal biofilms cannot develop with inhaled fungal conidia. In pathologic conditions, such as mucosal injury, exposure to air pollutants, or obstructed sinus ostium, can induce the development of fungal biofilms [5,6]. Biofilm formation in sinus mucosa could be an important factor in the development of recalcitrant chronic inflammatory disease.

Fungal rhinosinusitis used to be an uncommon disease. Recently, the prevalence of fungal rhinosinusitis, especially fungus balls, has continuously increased over time [7]. Most fungal sinusitis develops in healthy people and the risk factors for the development of fungal sinusitis are not completely understood. Fungal sinusitis most commonly occurs in the maxillary sinus followed by sphenoid sinus. The pathogenic theories for the development of fungal sinusitis are odontogenic, such as oroantral communication and endodontic treatment enhancing colonization of fungi in the sinus. Another possible cause is the overloading of fungal conidia through inhalation during respiration. However, most fungal sinusitis may develop when these two conditions work together [8]. Inspirated fungi in the maxillary sinus can grow with preexisting sinusitis or foreign bodies in the sinus. Fungal maxillary sinusitis is generally considered to be related to the inflammatory process associated with dental procedures [9]. The prevalence of maxillary sinusitis increases with an increasing aging population, and an increased number of endodontic treatments and dental implants. The metallic components used in endodontic treatments are speculated to be significant contributors to fungal sinusitis. Metallic contents with various cations tightly bound to polynucleotide and play a specific role in nucleic acid structure, function, and metabolism [10].

Metal ions show an important effect on fungal growth and their toxin synthesis. Zinc oxide (ZO), the main component of endodontic sealer, can promote fungal survival and proliferation [11]. During the endodontic treatment, root canal filling material can inadvertently be pushed into the maxillary sinus. The odontogenic theory of maxillary fungal sinusitis is primarily explained by the presence of ZO in endodontic sealer, which can cause an acute or chronic inflammatory process by paralysis of the epithelial cilia, mucosal edema, and hyperemia of the sinus mucosa [11]. The fungus ball contains various metallic components from different endodontic sealers, including ZO, and ZO can induce fungal growth in the maxillary sinus [8,11,12]. However, when the *Aspergillus fumigatus* were cultured with ZO on primary nasal epithelial cells, ZO did not influence *A. fumigatus*-induced nasal epithelial cell activation and fungal biofilm formation [13]. Furthermore, the immunopathologic role of ZO in the development of maxillary fungal sinusitis is not completely understood. In this study, the authors conducted experiments to determine whether the interaction between intramaxillary ZO and inhaled *A. fumigatus* could induce or exacerbate inflammatory response in the sinus mucosa and lead to the development of fungal sinusitis or ZO could not impact the influence of *A. fumigatus*-induced sinus mucosal inflammation.

## 2. Materials and Methods

### 2.1. Reagents

Tween-20 and nitrocellulose membranes were purchased from Bio-Rad (Hercules, CA, USA). ZO was obtained from Avention (AV-2014571, 99.9%, 10–20 nm/spherical, Incheon, Republic of Korea). Zoletil^®^ and Rompun^®^ for rabbit anesthesia were purchased from Virbac (Carros, France) and Bayer (Leverkusen, Germany), respectively. Enzyme-linked immunosorbent assay (ELISA) kits (Interleukin (IL)-1β, LS-F5127; IL-8, LS-F5371; tumor necrosis factor (TNF)-α, LS-F5368) and antibodies against c-JUN (LS-C368401), JNK (LS-C135466, and p38 (LS-C150443) were purchased from LSbio (Seattle, WA, USA). Trizol, LIVE/DEAD BacLight staining kit, the antibody against ERK (61-7400), and β-actin (AM4302) were acquired from Invitrogen (Carlsbad, CA, USA). SYBR Green PCR core kit was purchased from PE Applied Biosystems (Foster City, CA, USA). Lysis buffer was obtained from Thermo Fisher Scientific (Rockford, IL, USA). Antibodies against nuclear factor (NF)-κB (BS-0465R) were purchased from Bioss Antibodies Inc. (Woburn, MA, USA). Antibodies against peroxidase-conjugated anti-mouse (sc-516102) and anti-rabbit immunoglobulin G (sc-23570) were purchased from Santa Cruz Biotechnology (Santa Cruz, CA, USA).

### 2.2. Aspergillus fumigatus Conidia and ZO Preparation

*Aspergillus fumigatus* (ATCC 46645) was inoculated on potato dextrose/corn meal agar plates with cycloheximide for 5–7 days at 25 °C. Fungal conidia were collected according to a previously described method [14]. Briefly, conidia were collected by scrapping the plate with sterile phosphate buffer saline (PBS) that contained 0.05% tween-20. Eluates were centrifuged at 1000 rpm for 10 min, and pellet suspensions were filtered through a 40 μm cell strainer. Conidia suspensions at 2 × 10^7^/mL were placed at 45 °C to dry, and after that, they were stored at −80 °C until required.

ZO was dispersed in PBS at a concentration of 5 mg/mL and stored at −70 °C until further use in experiments.

### 2.3. Development of A. fumigatus-Induced Rhinosinusitis Rabbit Model

Pathogen-free male New Zealand white rabbits weighing 2.5 kg and 60 days old were used in this study (Hyosung Science Inc., Daegu, Republic of Korea). All rabbits were kept under standardized conditions for at least 1 week before the commencement of the experiments. This study was conducted in accordance with the guidelines of the National Institute of Health Guide for the Care and Use of Laboratory Animals and approved by the Institutional Review Board of Animal Experiments of Daegu Catholic University Medical Center (DCIAFCR-221129-36-Y).

A total of 28 rabbits were used for this study. Rabbits were divided into 4 different experimental groups. In group 1 (*n* = 6 sides each for 4 and 12 weeks), the negative control group in which rabbits were treated with intramaxillary PBS. Group 2 consisted of 8 sides each with intramaxillary PBS inoculation with ZO. Group 3 consisted of 8 sides each with intramaxillary inoculation *A. fumigatus* without ZO. Group 4 consisted of 8 sides each with intramaxillary *A. fumigatus* inoculation with ZO. Briefly, the animals were anesthetized with intramuscular injection of 17.5 mg/kg of Zoletil^®^ and 3.5 mg/kg of Rompun^®^. The nasal dorsum was prepared aseptically followed by local infiltration of lidocaine 2% with epinephrine 1:100,000. To expose the anterior wall of the maxillary sinus, the skin and subcutaneous tissues were dissected. The anterior wall of the maxillary sinus was drilled out (1.5 cm × 3.5 cm) without mucoperiosteal injury. The periosteal flap and skin were sutured using vicryl 4-0 and black nylon 4-0, respectively. PBS or 10^6^/100 μL of *A. fumigatus* conidia, and 5 mg/100 μL of ZO were inoculated into the maxillary sinus 3 times per week as needed. After 4 and 12 weeks, twenty-four hours after the last sinus inoculation, rabbits were euthanized with 100 mg/kg of thiopental sodium preceded by intramuscular sedation with Zoletil^®^ and Rompun^®^. After drilling out of the anterior wall of the maxillary sinus, sinus mucosa was collected for further study.

### 2.4. Measurement of Cytokines in Sinus Mucosa

Frozen sinus mucosa was homogenized and the IL-1β, IL-8, and TNF-α protein concentrations were determined using a commercially available ELISA kit. The specific sensitivities of the ELISA kit used were 7.8 pg/mL, 6.0 pg/mL, and 1.2 pg/mL for IL-1β, IL-8, and TNF-α (LS-F5368), respectively.

For quantitative reverse-transcription polymerase chain reaction (RT-PCR), Total RNA was extracted from the sinus mucosa using Trizol reagent. RNA purity and concentration were measured through a spectrophotometer (Beckman, Mountain View, CA, USA). Complementary DNA was made from 1 μg of RNA through amplification with a thermal cycler (PerkinElmer Corp., Norwalk, CT, USA). From the amplified cDNA, the quantitative PCR was performed using a SYBR Green PCR core kit. The primer sequences and amplification products were as follows: IL-1β sense 5′-GTCTTCCTAAAGCAAGCCTTAC-3′ and antisense 5′-GGGGTGTCACAATCTGTTTC-3′ (92 bp); IL-8 sense 5′-GCATAAAGACACACTCCACAC-3′ and antisense 5′-GTCCAGGCAGAGTTCTCTTC-3′, (131 bp); TNF-α sense 5′-CCTGTGCCTCCCTTCACTTAT-3′ and antisense 5′-TTTCTCGCCACTGACCAGTAG-3′ (157 bp), and GAPDH sense 5′-TAACTCTGGCAAAGTGGATGT-3′ and antisense 5′-CGTGGGTGGAATCATACTG-3′ (92 bp). Initial denaturation was performed at 95 °C for 2 min, followed by 40 cycles consisting of denaturation at 94 °C for 10 s, annealing at 60 °C for 10 s, and elongation at 72 °C for 45 s. All samples were amplified in triplicate. The expression levels of the aforementioned mRNA were normalized to the median value for GAPDH, and expression levels were measured using the relative quantification 2^−ΔΔCT^ method.

### 2.5. Measurement of Transcription Factors in Sinus Mucosa

The frozen sinus mucosa of each sample was homogenized, harvested, and lysed in an ice-cold lysis buffer. Whole-cell lysates were collected and subjected to sodium dodecyl sulfate polyacrylamide gel electrophoresis to separate proteins and were then transferred onto a nitrocellulose membrane. The membranes were blocked with 5% skim milk solution and were incubated with antibodies against NF-κB, c-JUN, JNK, p38, ERK, and β-actin. After 1 h of incubation, the membranes were washed with Tris-buffered saline with 0.1% Tween 20 and were then treated with peroxidase-conjugated anti-mouse antibody or anti-rabbit immunoglobulin G. Bands were visualized using horseradish peroxidase-conjugated secondary antibodies and an enhanced chemiluminescence system (Pierce, Rockford, IL, USA). Band densities were measured using the multi Gauge v.2.02 software (Fujifilm, Tokyo, Japan) and were expressed as a percentage of treated versus untreated cells.

### 2.6. Histological Analysis of Sinus Mucosa

The tissues were fixed with 10% formalin solution and embedded in paraffin for sectioning at 5 μm intervals. Inflammatory cell infiltration and epithelial thickness were quantified in hematoxylin and eosin (H&E) stained sections. Eosinophil and neutrophil infiltration were calculated as the average number of cells in five high-power fields. Goblet cell hyperplasia was determined by periodic acid-Schiff (PAS) staining, and the average number of goblet cells was counted at ×200 magnification. Epithelial and subepithelial thickness was directly measured at ×400 magnification through a video camera (Olympus Optical Co., Ltd., Tokyo, Japan) and analyzed using the DP Controller software (ver. 2.2.1.227). All tissue sections were blindly examined in terms of tissue origin, and the mean counts were determined at five different mucosal areas.

### 2.7. Biofilm Detection in Sinus Mucosa

Rabbit sinus mucosa was washed thoroughly with normal saline and then incubated in 0.1 mL LIVE/DEAD BacLight staining mixture at room temperature in darkness for 15 min as reported [15]. Viable cells and fungi stained green, while damaged cells and fungi stained red. Biofilms were observed with confocal scanning laser microscopy. Typical fungal biofilm fluorescence was observed when fluorescing areas displayed a highly coordinated network of hyphae, using a Nikon A1 confocal microscope (Nikon, Tokyo, Japan).

### 2.8. Statistical Analysis

A statistical Package for the Social Sciences version 25.0 (IBM Corp., Armonk, NY, USA) was used to analyze data obtained in the experiments, which were presented as the mean ± standard deviation. One-way analysis of variance (ANOVA) followed by Tukey’s test was performed for normally distributed data and the Kruskal–Wallis test with post-hoc Bonferroni–Dunn test was performed for non-normally distributed data. A *p*-value of <0.05 was considered statistically significant.

## 3. Results

### 3.1. Effect of A. fumigatus Conidia and ZO on Cytokine Protein and mRNA Levels in Sinonasal Mucosa

After 12 weeks, only 1 of 8 cases in *A. fumigatus* alone inoculation group and 2 of 8 cases in ZO with *A. fumigatus* inoculation group had mucoid secretions in the maxillary sinus. Other rabbits’ maxillary sinus cavities looked good in the negative control group. And they did not show any other sinusitis symptoms, such as runny nose or breathing difficulty.

**Figure 1:**
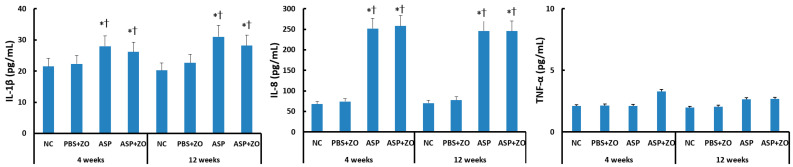
Interleukin (IL)-1β, IL-8, and tumor necrosis factor (TNF)-α protein levels in sinus mucosa of rabbits. IL-1β and IL-8 protein levels were significantly increased by intramaxillary instillation of *Aspergillus fumigatus* (ASP) with or without Zinc oxide (ZO) at 4 and 12 weeks. NC; negative control, PBS; phosphate buffer saline, * *p* < 0.05 compared with NC, † *p* < 0.05 compared with PBS + ZO or ASP groups.

The protein levels of IL-1β, IL-8, and TNF-α in the sinus mucosa were determined using the ELISA method. IL-1β and IL-8 protein levels were significantly increased by intramaxillary instillation of *A. fumigatus* after 4 weeks (IL-1β; 28.0 ± 8.5 pg/mL, IL-8; 252.1 ± 86.2 pg/mL) and 12 weeks (IL-1β; 31.0 ± 11.6 pg/mL, IL-8; 246.1 ± 96.7 pg/mL). However, the intramaxillary presence of ZO did not influence IL-1β (26.2 ± 12.5 pg/mL, 28.2 ± 13.6 pg/mL, respectively) and IL-8 (258.3 ± 112.6 pg/mL, 245.6 ± 128.8 pg/mL, respectively) protein levels in sinus mucosa. When comparing 4 weeks and 12 weeks, experiment duration did not influence IL-1β and IL-8 protein levels. Intramaxillary *A. fumigatus* and ZO did not influence TNF-α protein level in the sinus mucosa (Figure 1).

**Figure 2:**
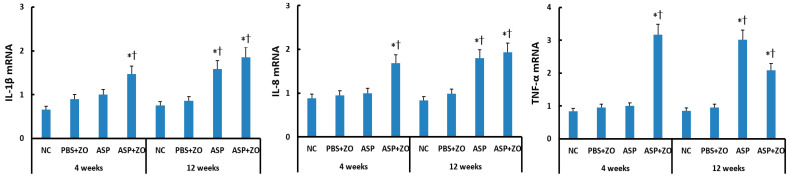
Interleukin (IL)-1β, IL-8, and tumor necrosis factor (TNF)-α mRNA expression in sinonasal mucosal of rabbits. Intramaxillary instillation of *Aspergillus fumigatus* (ASP) with ZO enhanced IL-1β, IL-8, and TNF-α mRNA expression at 4 and 12 weeks. These cytokine mRNA expressions were also increased at 12 weeks without ZO. NC; negative control, PBS; phosphate buffer saline, * *p* < 0.05 compared with NC, † *p* < 0.05 compared with PBS + ZO or ASP groups.

The mRNA levels of IL-1β, IL-8, and TNF-α in the sinus mucosa were determined using RT-PCR. When comparing with the housekeeping gene, intramaxillary instillation of *A. fumigatus* with ZO significantly enhanced IL-1β, IL-8, and TNF-α mRNA expression at 4 and 12 weeks. Intramaxillary instillation of *A. fumigatus* without ZO, only enhanced these mRNA expressions at 12 weeks. When comparing 4 weeks and 12 weeks, experiment duration did not influence IL-1β, IL-8, and TNF-α mRNA expression. Although the intramaxillary presence of ZO enhanced mRNA expressions at 4 weeks, it did not influence IL-1β, IL-8, and TNF-α mRNA expressions at 12 weeks (Figure 2).

### 3.2. Effect of A. fumigatus Conidia and ZO on Transcription Factor Expression in Sinonasal Mucosa

**Figure 3:**
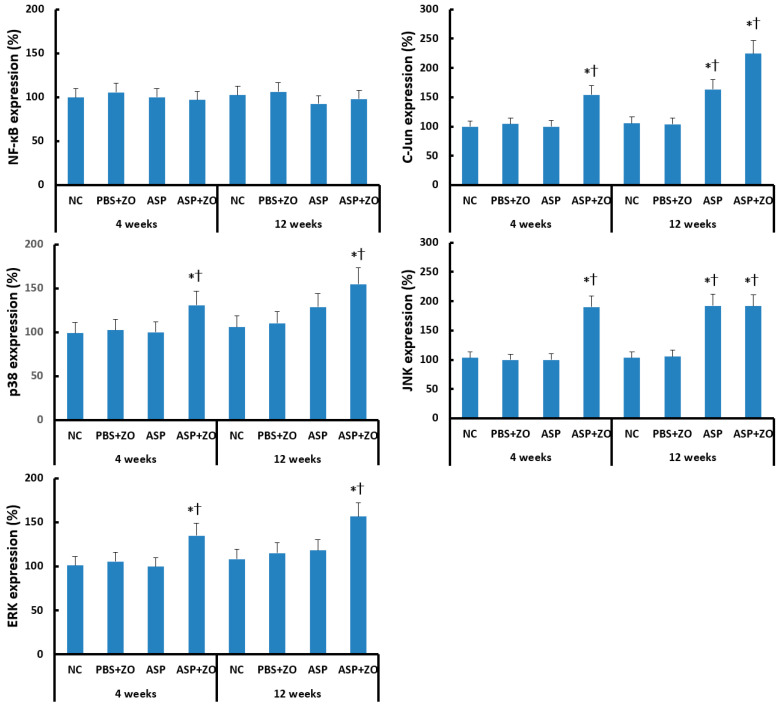
Transcription factors, nuclear factor (NF)-κB, C-Jun, p38, JNK, and ERK expression in sinus mucosa. Intramaxillary (Intra) instillation of *Aspergillus fumigatus* (ASP) and zinc oxide (ZO) significantly increased C-Jun, p38, JNK, and ERK expression at 4 and 12 weeks. However, *A. fumigatus* alone enhanced C-Jun and JNK expression at 12 weeks. NC; negative control, PBS; phosphate buffer saline, * *p* < 0.05 compared with NC, † *p* < 0.05 compared with PBS + ZO or ASP groups.

Transcription factor expression in sinus mucosa was determined using Western blot analysis. Intramaxillary instillation of *A. fumigatus* with ZO significantly enhanced C-Jun, p38, JNK, and ERK expression at 4 and 12 weeks. Intramaxillary instillation of *A. fumigatus* without ZO enhanced C-Jun and JNK expression at 12 weeks. Intra maxillary presence of ZO did not enhance C-Jun, JNK expression at 12 weeks. Intramaxillary instillation of *A. fumigatus* or ZO did not influence NF-κB expression in sinus mucosa (Figure 3).

### 3.3. Effect of A. fumigatus Conidia and ZO on Histologic Changes in Sinonasal Mucosa

**Figure 4:**
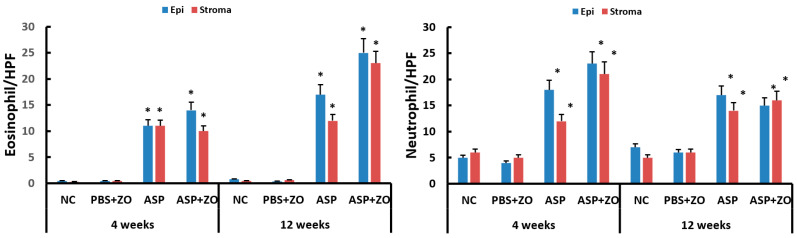
Number of eosinophils and neutrophils in sinus mucosa. Intramaxillary instillation (Intra) of *Aspergillus fumigatus* (ASP) with or without zinc oxide (ZO) significantly increased both eosinophil and neutrophil infiltration in epithelial (Epi) and stroma layer. NC; negative control, PBS; phosphate buffer saline, * *p* < 0.05 compared with NC or PBS + ZO groups.

Eosinophil and neutrophil infiltration in epithelial and subepithelial layers were determined using light microscopy. The number of eosinophils and neutrophils in sinus mucosa was significantly increased with intramaxillary instillation of *A. fumigatus* with or without ZO at 4 and 12 weeks compared to negative control or PBS with ZO group. Although it was not statistically significant, the number of eosinophils was increased in a time-dependent manner. The presence of intramaxillary ZO did not influence eosinophil and neutrophil infiltration (Figure 4).

**Figure 5:**
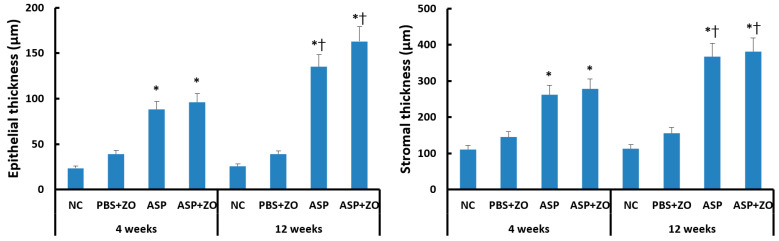
Epithelial and stromal thickness in sinus mucosa. Intramaxillary instillation (Intra) of *Aspergillus fumigatus* (ASP) with or without zinc oxide (ZO) significantly increased epithelial and stromal thickness both at 4 and 12 weeks. These thicknesses were significantly increased at 12 weeks in comparison with 4 weeks. NC; negative control, PBS; phosphate buffer saline, * *p* < 0.05 compared with NC or PBS + ZO groups, † *p* < 0.05 compared with 4 weeks.

Intramaxillary instillation of *A. fumigatus* with or without ZO significantly induced epithelial and stromal thickening compared to negative control or PBS with ZO group. After 12 weeks of treatment with *A. fumigatus* with or without ZO (3.5–6.4 times compared to control groups), the epithelial thickness was much increased in comparison with 4 weeks groups (2.3–4.1 times compared to control groups). Stromal thickness also significantly increased at 12 weeks (2.4–3.4 times compared to control groups) in comparison with 4 weeks (1.8–2.5 times compared to control groups) (Figure 5). However, the presence of intramaxillary ZO did not influence epithelial and stromal thickness.

**Figure 6:**
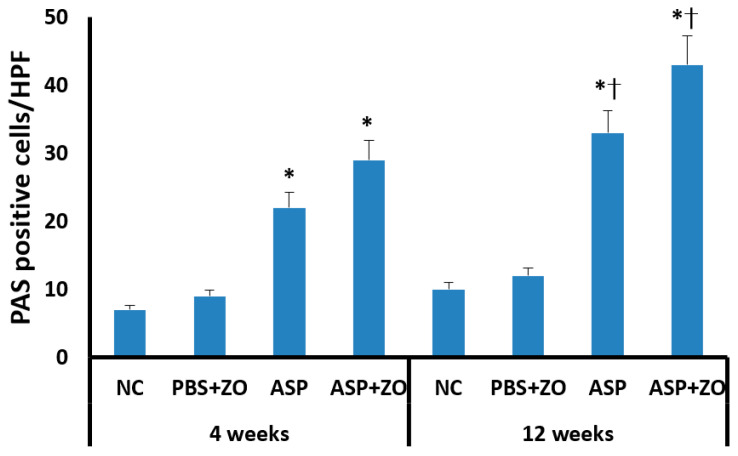
The number of periodic acid-Schiff positive (PAS) cells in sinus mucosa. Intramaxillary instillation (Intra) of *Aspergillus fumigatus* (ASP) with or without zinc oxide (ZO) significantly increased PAS-positive goblet cells both at 4 and 12 weeks. PAS-positive cells were significantly increased at 12 weeks in comparison with 4 weeks. NC; negative control, PBS; phosphate buffer saline, * *p* < 0.05 compared with NC or PBS + ZO groups, † *p* < 0.05 compared with 4 weeks.

Secreting goblet cell numbers were quantified using a PAS stain. PAS-positive goblet cells in sinus mucosa were significantly increased in the intramaxillary instillation of *A. fumigatus* with or without ZO groups compared to negative control and PBS with ZO groups. After 12 weeks of treatment with *A. fumigatus* with (43.2 ± 19.7/HPF) or without ZO (33.4 ± 13.6/HPF), the number of PAS-positive cells was much higher in comparison with 4 weeks groups (*A. fumigatus* alone; 21.7 ± 8.6/HPF, *A. fumigatus* with ZO; 29.4 ± 9.2/HPF) (Figure 6). However, the presence of intramaxillary ZO did not significantly influence the number of PAS-positive cells.

### 3.4. Effect of ZO on Development of A. fumigatus Biofilm on Sinonasal Mucosa

**Figure 7:**
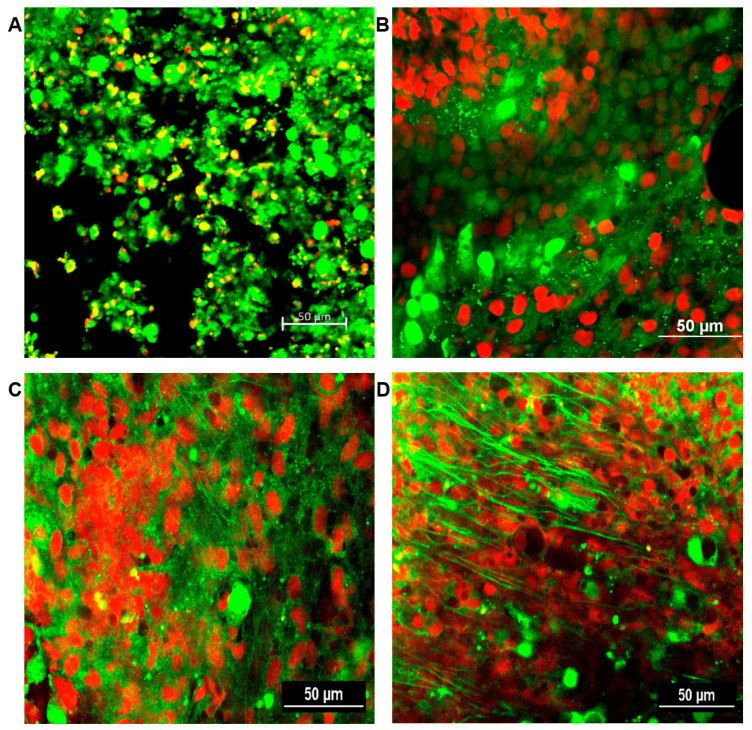
Representative confocal scanning microscopic findings in sinus mucosa of experimental groups after 12 weeks. Healthy cells and fungi with intact membranes were stained green, whereas those with damaged membranes stained red. (**A**) negative control; (**B**) phosphate buffer saline with zinc oxide (ZO); (**C**) *Aspergillus fumigatus* alone; (**D**) *A. fumigatus* with ZO. 20× magnification, scale bars = 50 μm.

The formation of fungal biofilms was determined for all sinus mucosal samples. A confocal laser scanning microscope can distinguish live and dead cells with LIVE/DEAD BacLight staining. Fungal biofilms were observed as strong green fluorescent displayed highly coordinated network of hyphae, spreading in all directions and crosslinking each other with metabolically active cells. No fungal biofilm was detected in the sinus mucosa of negative control and PBS with ZO groups. From 4 weeks, intramaxillary inoculation of *A. fumigatus* with or without ZO showed fungal biofilms with increased green fluorescence in every sinus mucosa. Although, *A. fumigatus* with ZO groups revealed thicker green clusters formation, no significant difference could be objectively demonstrated between the two groups (Figure 7).

## 4. Discussion

The maxillary sinus is the most commonly involved fungal sinusitis, followed by sphenoid and frontal sinus. Maxillary molars and premolars are closely located with the maxillary sinus floor and can cause the spread of odontogenic inflammation to the maxillary sinus. So, the odontogenic treatment of the maxillary teeth can develop maxillary sinusitis [16]. Endodontic sealers can enter into the maxillary sinus during endodontic treatment and can influence the development of fungal sinusitis. In this study, we tried to elucidate the immunopathologic role of ZO, the main component of endodontic sealer, in the development of fungal maxillary sinusitis. Acute rhinosinusitis, which involves sinonsal inflammation lasting less than 4 weeks, and CRS, characterized by the presence of sinonasal symptoms for longer than 12 weeks, exhibit different immunopathologic mechanisms [17]. To compare acute and chronic immunopathologic responses induced by *A. fumigatus* or ZO, we evaluated mucosal inflammatory responses and biofilm formation at 4 and 12 weeks. Although intramaxillary instillation of *A. fumigatus* conidia enhanced the inflammatory process of sinus mucosa, the presence of ZO did not significantly influence the mucosal inflammation.

Rabbits have been widely used in research on sinus diseases, especially maxillary sinus, due to their anatomical similarity to humans and enough size for experiments [18]. The average temperature of rabbit maxillary sinuses, 35.2 ± 0.7 °C, provides favorable conditions for the growth of bacteria and fungi [19]. The obstruction of the sinus natural ostium or proceeding sinus mucosal injury along with the administration of 10^8^/mL of *A. fumigatus* conidia into the maxillary sinus could induce the development of fungal sinusitis with visible mucopurulent discharge [19]. In the air, 50–50,000 spores/m^3^ are present, and these are continuously inhaled into the airway. The number of spores that enter the airway can vary widely depending on individual circumstances, and only a few studies have directly measured the deposition of conidia within the airway [20]. Fungal airway disease can develop even at low concentrations of fungi, and determining the exact minimal fungal concentration required to develop fungal sinusitis is difficult. In contrast to a previous study, which used 10^8^/mL of conidia with mucosal injury or closure of the sinus ostium, we induced sinus inflammation by administering 10^7^/mL of conidia without mucosal injury or ostium closure. We could not observe gross evidence of sinusitis, such as purulent discharge, only mucoid discharge was found in 1 or 2 cases in groups 3 and 4. *A. fumigatus* was cultured in 87.5% (7 out of 8) in groups 3 and 4 using potato dextrose/corn meal agar culture plates at 4 and 12 weeks. Intramaxillary instillation of *A. fumigatus* conidia could develop fungal biofilms in the sinus mucosa. This suggests that continuous exposure of the sinus mucosa to fungi leads to the growth and proliferation of fungi within the sinus. *A. fumigatus* conidia contains sialic acid residues and facilitates binding to basal lamina protein of nasal epithelial cells [21]. The germination of conidia and filamentous growth of fungi with the interaction of polysaccharides in the extracellular matrix can develop biofilms. *A. fumigatus* can form biofilm on primary nasal epithelial cells and various hazardous conditions can exacerbate the development of fungal biofilms [6,14]. ZO can paralyze the mucociliary function or cause edema and hyperemia of sinus mucosa, which induce a favorable condition for fungal growth and the development of fungal biofilms [8,11]. However, the addition of ZO did not affect *A. fumigatus* biofilm formation when *A. fumigatus* was cultured on primary human nasal epithelial cells [13]. Although we could not quantify the fungal biofilm formation and difficult to determine whether intramaxillary ZO affects *A. fumigatus* biofilm formation, fungal biofilms were observed at 4 and 12 weeks using a confocal microscope.

The human body has a defense against fungi through innate cell-mediated immunity through the phagocytic activity of macrophages, neutrophils, and Th1 lymphocytes [22]. However, an enhanced role of Th2 lymphocytes can lead to fungal infections. In this study, the administration of *A. fumigatus* into the maxillary sinus increases both Th1 and Th2 inflammatory cell infiltration in sinus mucosa. This indicates that *A. fumigatus* simultaneously induces the activation of both defense mechanisms and inflammatory responses against fungi in sinus mucosa. Fungal protease can induce not only direct damage to the epithelial physical barrier but also airway inflammation and cytokine production through interaction with pattern recognition receptors of epithelial cells [23,24]. *A. fumigatus* enhanced the expression of IL-1β, IL-8, and TNF-α mRNA and promoted the production of IL-1β and IL-8 proteins in maxillary sinus mucosa. Despite the increase in TNF-α mRNA, TNF-α protein production did not increase, suggesting that the administered fungi may not have sufficiently induced local inflammatory reactions. IL-1β is a proinflammatory cytokine that acts as a potent mediator of the inflammatory response through the stimulation and promotion of inflammatory cell migration. IL-8 also acts as a proinflammatory cytokine and chemokine, which contributes to the initiation and amplification of inflammatory responses and migration of neutrophils. ZO has been known as an indispensable component for fungal growth and activation for fungal metabolism [11]. ZO could increase the amount of fungi in contact with epithelial cells by acting as a nidus for fungal growth and interfering with the mucociliary movement of epithelial cells, thereby prolonging the contact time between the fungi and the epithelial cells. This leads to the maintenance and exacerbation of the inflammatory responses in the sinonasal mucosa [11,25]. Although, intramaxillary instillation of ZO enhanced IL-1β, IL-8, and TNF-α mRNA expressions at 4 weeks of experiment, after 12 weeks ZO did not influence both mRNA expression and protein production from sinus mucosa. Fungi induce the production of chemical mediators through the expression of various transcription factors [26]. *A. fumigatus* enhanced expression of mitogen-activated protein kinase and activator protein 1 in sinus mucosa. These transcription factor expression patterns were similar to chemical mediator expression at 4 and 12 weeks. ZO also did not influence transcription factor expression in sinus mucosa.

Metallic materials of endodontic treatment can develop maxillary sinus fungal disease [9,27]. ZO, the main component of endodontic sealer, could play an important role in the development of fungal maxillary sinus diseases. In this study, intramaxillary ZO did not influence *A. fumigatus*-induced sinus mucosal inflammation. Based on the results of this study, it seems difficult to make a conclusion on whether ZO can exacerbate fungi-induced sinus mucosal inflammation. In this study, 10^7^/mL of *A. fumigatus* conidia were administered three times a week. It was based on the previous in vitro studies, which reported that 1 × 10^4^/mL to 1 × 10^6^/mL conidia are sufficient for fungal biofilm formation without cytotoxic effect [28]. However, it is possible that the number of conidia is not enough to induce sinusitis in vivo study. Additionally, the fungal conidia were administered in the maxillary sinus, the rabbit without sinus mucosal injury or obliteration of sinus ostium, which possibly led to the removal of a significant portion of the administered fungal conidia by the mucociliary clearance system. Dufour et al. suggested that obstruction of the sinus ostium appears to play a major role in the pathogenesis of fungal sinusitis [19]. In the aspect of ZO, we used 5 mg/100 μL. Administered concentration of ZO may not be sufficient to exacerbate fungi-induced mucosal inflammation or ZO also can be removed by mucociliary action of sinus mucosa. Moreover, the frequency of administration (three times a week) or the experimental duration may not have been adequate to induce fungal sinusitis. Generally, it takes several years for a fungus ball to develop after endodontic treatment [11]. Although intramaxillary ZO did not influence the expression of inflammatory cytokines and their transcription factors, ZO did enhance histological change in sinonasal mucosa, such as epithelial and stromal thickness and the number of mucus-producing cells, in a time-dependent manner. These histological changes suggest that extending the experimental period may allow ZO to also impact mucosal inflammation. The relatively small experimental sample size used in this study likely acted as a limitation in analyzing the impact of ZO on the development of fungal rhinosinusitis in the rabbit model. To accurately understand the impact of ZO on the development of fungal sinusitis, further studies utilizing various concentrations of fungal conidia and ZO over an extended period may be necessary.

## 5. Conclusions

ZO has been known as a risk factor for the development of fungal sinusitis. We conducted this study to determine whether the presence of ZO within the maxillary sinus would influence or exacerbate *A. fumigatus*-induced sinus mucosal inflammation. Intramaxillary administration of *A. fumigatus* conidia induced an inflammatory response by enhancing the expression of IL-1β, IL-8, and TNF-α and promoting biofilm formation in the sinus mucosa. Intramaxillary ZO did not exacerbate the inflammatory response induced by the fungus. However, ZO significantly influenced histological change (epithelial and stromal thickness, and the number of mucus-producing cells) in the sinonasal mucosa in a time-dependent manner. This discrepancy may be attributed to factors such as the quantity of fungal conidia, the concentration of administered ZO used in this study, and the pathophysiological condition of the maxillary sinus, which could have influenced the results. Therefore, further study is needed to explore the association between ZO and fungal inflammatory diseases.

## Data Availability

Data supporting this study can be obtained by contacting the corresponding author.
